# Dominant Role of PI3K p110α over p110β in Insulin and β-Adrenergic Receptor Signalling

**DOI:** 10.3390/ijms222312813

**Published:** 2021-11-26

**Authors:** Biqin Zhang, Cheukyau Luk, Joyce Valadares, Christos Aronis, Lazaros C. Foukas

**Affiliations:** Institute of Healthy Ageing, Department of Genetics, Evolution and Environment, University College London, London WC1E 6BT, UK; biqin.zhang.19@ucl.ac.uk (B.Z.); umcyl@leeds.ac.uk (C.L.); joycemv6@gmail.com (J.V.); chrisaronis@gmail.com (C.A.)

**Keywords:** phosphoinositide 3-kinase, insulin receptor, insulin signalling, β-adrenergic receptor, adrenergic signalling, insulin resistance, obesity, type 2 diabetes

## Abstract

Attribution of specific roles to the two ubiquitously expressed PI 3-kinase (PI3K) isoforms p110α and p110β in biological functions they have been implicated, such as in insulin signalling, has been challenging. While p110α has been demonstrated to be the principal isoform activated downstream of the insulin receptor, several studies have provided evidence for a role of p110β. Here we have used isoform-selective inhibitors to estimate the relative contribution of each of these isoforms in insulin signalling in adipocytes, which are a cell type with essential roles in regulation of metabolism at the systemic level. Consistent with previous genetic and pharmacological studies, we found that p110α is the principal isoform activated downstream of the insulin receptor under physiological conditions. p110α interaction with Ras enhanced the strength of p110α activation by insulin. However, this interaction did not account for the selectivity for p110α over p110β in insulin signalling. We also demonstrate that p110α is the principal isoform activated downstream of the β-adrenergic receptor (β-AR), another important signalling pathway in metabolic regulation, through a mechanism involving activation of the cAMP effector molecule EPAC1. This study offers further insights in the role of PI3K isoforms in the regulation of energy metabolism with implications for the therapeutic application of selective inhibitors of these isoforms.

## 1. Introduction

Class I PI3Ks is a family of enzymes involved in transmission of signals from diverse cell surface receptors [1]. Class I PI3K consists of four catalytic isoforms: p110α, p110β, p110γ and p110ẟ. Of these isoforms, p110α and p110β are ubiquitously expressed. So far, attribution of specific functions to each of p110α and p110β has been challenging and occasionally controversial. Genetic and pharmacological studies have demonstrated that p110α is the principal isoform activated downstream of the insulin receptor [2,3]. The prominent role of p110α in insulin signalling has been shown to correlate with selective recruitment of p110α in insulin receptor substrate-1 (IRS-1) complexes [2]. However, other studies have reported metabolic phenotypes occurring from targeting the gene encoding p110β in mice. Mice globally targeted at the gene encoding p110β displayed growth retardation and developed mild insulin resistance with age [4]. Moreover, mice with liver-specific deletion of p110β showed impaired insulin sensitivity and glucose tolerance [5]. More recently, another study demonstrated that p110β can substitute for p110α in insulin-stimulated Akt phosphorylation in hepatocytes from liver-specific p110α-deleted mice [6]. On this basis, the study concluded that p110α and p110β are redundant in insulin signalling in hepatocytes.

In addition to the prominent and well-established role of PI3Ks in insulin signalling, PI3Ks have also been implicated in adrenergic signalling. It has been shown that β3-AR agonists can stimulate Akt activation in a wortmannin-sensitive manner in adipocytes [7]. More recently, p110α has been implicated in Akt stimulation downstream of the β-AR [8]. However, the mechanism of activation of p110α by β-AR remains unknown. Furthermore, whether p110β also transmits signals downstream of the β-AR has not been addressed.

PI3Ks p110α and p110β have been pursued as therapeutic targets mainly in oncology [9]. However, inhibition of p110α has recently been shown to increase energy expenditure through potentiation of β-AR signalling and enhancement of mitochondrial activity in adipocytes [8,10]. The potentiating effect of p110α inhibition in β-AR signalling has also been shown in human adipose tissue [11]. Furthermore, p110α inhibition has been shown to reduce adiposity and metabolic syndrome in mice and rhesus monkeys [12,13]. These findings open the possibility of targeting these isoforms in obesity and associated diseases in the future. Knowledge of the precise contribution of each of these isoforms in metabolic pathways is therefore very important for assessment of on-target and off-target effects of these isoforms’ inhibition.

In the present study, we have revisited the question of the relative contribution of each of the p110α and p110β isoforms to insulin receptor signalling. We have also sought to dissect the molecular mechanism of PI3K activation by β-AR. For this purpose, we have used murine brown adipocytes from wild-type or adipose tissue-specific p110α deleted mice as well as 3T3-L1 adipocytes in conjunction with treatment with p110α- and p110β-selective inhibitors. Our data support the previously demonstrated dominance of p110α over p110β in insulin receptor signalling. Consistent with a previous study which demonstrated that functional Ras is required for maximal p110α activation by insulin [6], we show that embryonic fibroblasts from mice with p110α-Ras binding domain (RBD) mutations that impair the interaction of p110α with Ras, have reduced insulin-stimulated Akt phosphorylation. However, Ras binding does not account for the selectivity for p110α over p110β in insulin signalling. Finally, we show that p110β has no substantial contribution to β-AR stimulation of Akt and that the cAMP-activated guanine nucleotide exchange factor (GEF) EPAC1 is a key mediator in the mechanism of p110α activation by β-AR. These studies further clarify the roles of the ubiquitously expressed PI3K isoforms in key metabolic pathways and add important information in light of the ongoing efforts for therapeutic application of PI3K isoform-selective inhibitors.

## 2. Results

### 2.1. p110α Is Preferentially Engaged over p110β in Insulin Signalling

**Figure 1:**
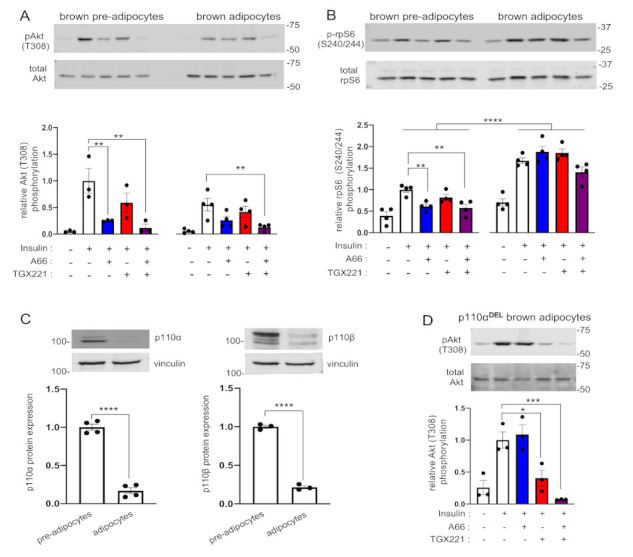
p110α is the principal PI3K isoform engaged in insulin signalling in brown adipocytes. Brown pre-adipocytes or differentiated adipocytes were treated with A66 (1 μM, blue graph bars) or TGX221 (0.5 μM, red graph bars) or a combination of both inhibitors (purple graph bars) followed by stimulation with 100 nM of insulin for 15 min at 37 °C. (**A**) Levels of Akt (T308) phosphorylation were determined by immunoblot analysis. Phosphorylation levels were normalised to total Akt levels detected in a second blot performed in parallel using the same lysates. (**B**) Ribosomal protein S6 (rpS6) (S240/244) phosphorylation was also detected on the same immunoblots. (**C**) The levels of expression of p110α and p110β in pre-adipocytes and differentiated adipocytes used in the signalling experiments were also determined by immunoblot analysis. p110α and p110β signal intensities were normalised to vinculin (used as a loading control). (**D**) Akt (T308) phosphorylation in p110α-deficient (p110α^DEL^) brown adipocytes treated and stimulated as above. Representative immunoblots and bar graphs with data from three or four (*n* = 3–4) independent experiments are shown. Data are presented as mean ± SEM. Statistical analysis was performed by one-way ANOVA with Dunnett’s multiple comparisons test between insulin-stimulated vehicle-treated and insulin-stimulated inhibitor-treated samples (**A**,**B**,**D**) or by unpaired two-tailed *t*-test (**C**). Comparisons between pre-adipocytes and differentiated adipocytes in (**B**) were performed by two-way ANOVA with Sidak’s multiple comparisons test. * *p* < 0.05; ** *p* < 0.01; *** *p* < 0.001; **** *p* < 0.0001.

In order to examine the relative contribution of each of the p110α and p110β isoforms in signalling downstream of the insulin and β-adrenergic receptors, we chose to use adipocytes as both of these pathways are critical in the biology of this cell type. We used immortalised adipocytes treated with the p110α- and p110β-selective inhibitors A66 and TGX221, respectively, and stimulated with insulin. The A66 and TGX221 inhibitors have extensively been characterised before and shown to be largely specific up to the concentration of 10 μM [14]. We have chosen doses within a range shown before to be effective in inhibiting insulin signalling while maintaining isoform selectivity [14,15]. We performed treatments in both pre-adipocytes (fibroblasts) and differentiated adipocytes to test whether the differentiation state affects response to inhibitors. We measured Akt phosphorylation and ribosomal protein S6 (rpS6) phosphorylation (a readout of S6-kinase (S6K) activation) to assess proximal and distal signalling pathway activation downstream of the insulin receptor. Consistent with a prominent role of p110α in insulin receptor signalling, treatment with A66 inhibited insulin-stimulated Akt phosphorylation by approximately 75% in pre-adipocytes and 53% in differentiated adipocytes, respectively (Figure 1A). TGX221 showed a tendency for an inhibitory effect that did not reach statistical significance, although concomitant treatment with TGX221 increased the inhibitory effect of A66. Similar to Akt, phosphorylation of rpS6 was consistently reduced only in A66-treated brown pre-adipocytes (Figure 1B). Interestingly, although insulin-stimulated rpS6 phosphorylation was higher in differentiated adipocytes compared to pre-adipocytes, it was insensitive to either inhibitor. Although the tested phosphorylation site (S240/244) is thought to be specific for S6K [16], it is possible that other insulin-stimulated kinases might phosphorylate this site in a PI3K-independent manner in this cell type under our experimental conditions. Consistent with this possibility, rapamycin-insensitive phosphorylation of rpS6 in S4240/244 has been reported before in IMR-90 human fibroblasts [17]. Another interesting observation was that ex vivo differentiation of brown pre-adipocytes to mature adipocytes reduced protein expression levels of both p110α and p110β to a great extent (Figure 1C). We also performed the same experiment in brown adipocytes isolated from mice with adipose tissue-specific p110α deletion. We found that insulin-stimulated Akt phosphorylation in adipocytes with p110α deletion was insensitive to A66 and was instead sensitive to TGX221 (Figure 1D). Combined treatment with A66 and TGX221 had a stronger inhibitory effect than either single treatment likely due to a residual p110α activity from incomplete gene deletion in some of the cells of the brown adipocyte pools. Our findings in adipocytes with p110α deletion are consistent with the recently reported role for p110β in insulin signalling in hepatocytes lacking p110α [6] and demonstrate that p110β has the ability to substitute for p110α in mediating insulin-stimulated Akt phosphorylation in the absence of the p110α protein.

In order to complement our pharmacological experiments, we also used MEFs with a p110β kinase-dead (D931A) mutation. As shown in Figure S1, lack of catalytically active p110β did not impair insulin-stimulated Akt phosphorylation. On the contrary, p110β^D931A^ MEFs displayed higher insulin-stimulated Akt phosphorylation compared to control MEFs. These data are consistent with p110α being the principal insulin activated PI3K isoform in this cell type as well and genetically corroborate the results obtained with use of the isoform-selective inhibitors.

**Figure 2:**
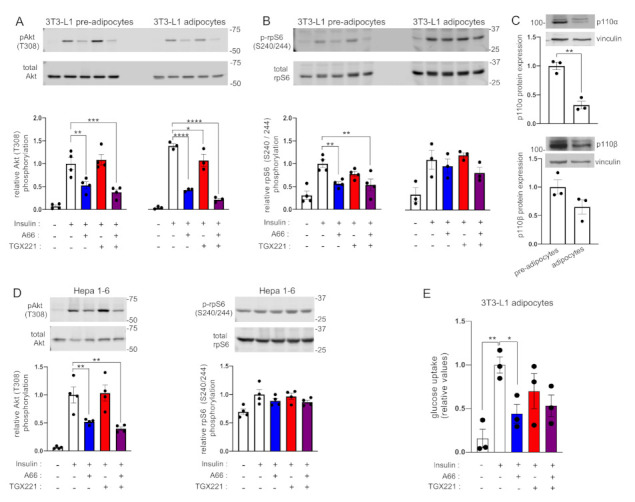
p110α is the principal PI3K isoform engaged in insulin signalling in 3T3-L1 adipocytes and Hepa 1.6 cells. 3T3-L1 pre-adipocytes or differentiated adipocytes or murine Hepa 1.6 hepatocytes were treated with A66 (1 μM, blue graph bars) or TGX221 (0.5 μM, red graph bars) or a combination of both inhibitors (purple graph bars) followed by stimulation with 100 nM of insulin for 15 min at 37 °C. (**A**) Levels of Akt (T308) phosphorylation in 3T3-L1 pre-adipocytes and adipocytes were determined by immunoblot analysis. Phosphorylation levels were normalised to total Akt levels detected in a second blot performed in parallel using the same lysates. (**B**) Ribosomal protein S6 (rpS6) phosphorylation (S240/244) was also detected in the same immunoblots. (**C**) The levels of expression of p110α and p110β in pre-adipocytes and differentiated 3T3-L1 adipocytes used for the signalling experiments were also determined by immunoblot analysis. (**D**) Akt (T308) and rpS6 (S240/244) phosphorylation in Hepa 1-6 murine hepatoma cells treated and stimulated as above. Representative immunoblots and bar graphs with pooled data from three or four (*n* = 3–4) independent experiments are shown. Data are presented as mean ± SEM. (**E**) Insulin-stimulated glucose uptake in 3T3-L1 adipocytes treated with inhibitors as above. Data from three (*n* = 3) independent experiments are shown. Statistical analysis was performed by one-way ANOVA with Dunnett’s multiple comparisons test between insulin-stimulated vehicle-treated and insulin-stimulated inhibitor-treated samples (**A**,**B**,**D**,**E**) or by unpaired two-tailed *t*-test (**C**). * *p* < 0.05; ** *p* < 0.01; *** *p* < 0.001; **** *p* < 0.0001.

We also performed similar experiments in murine 3T3-L1 adipocytes. In line with our findings in brown adipocytes, insulin-stimulated Akt was consistently inhibited by A66 in both 3T3-L1 fibroblasts and differentiated adipocytes (Figure 2A). TGX221 inhibited Akt phosphorylation by 24%, compared to 69% by A66, only in differentiated 3T3-L1 adipocytes. Moreover, rpS6 (S240/244) phosphorylation was sensitive to A66 only in 3T3-L1 pre-adipocytes, but insensitive to both A66 and TGX221 in ex vivo differentiated adipocytes (Figure 2B). Similar to brown adipocytes, both p110α and p110β protein expression was reduced in differentiated 3T3-L1 adipocytes compared to pre-adipocytes (Figure 2C). This finding was in contrast to the previously reported upregulation of p110β upon 3T3-L1 differentiation, which had been proposed as an indication of a key role of p110β in insulin signalling [18]. As it has been claimed that p110α and p110β have redundant roles in insulin signalling in hepatocytes [6], we also tried the same experiment using the Hepa 1-6 murine hepatoma cell line. We found that A66 inhibited Akt phosphorylation by 50%, whereas combined treatment with A66 and TGX221 only marginally reduced phosphorylation below that from A66 (Figure 2D). This might be explained by possible engagement of the p110δ isoform in insulin signalling in hepatoma cells, as an earlier study had demonstrated that inhibition of the p110δ isoform in addition to p110α was required for substantial inhibition of insulin-stimulated Akt phosphorylation in HepG2 hepatoma cells [19]. Similar to brown and 3T3-L1 differentiated adipocytes, rpS6 (S240/244) phosphorylation was insensitive to p110α and p110β inhibition in Hepa 1-6 cells. Finally, in order to further ascertain the prominence of p110α in insulin receptor signalling, we tested the effect of the inhibitors on glucose uptake, a functional output of insulin action in 3T3-L1 adipocytes. We found that only A66 inhibited insulin-stimulated glucose uptake (Figure 2E). These findings are consistent with previous data showing that only p110α inhibition diminished glucose uptake in 3T3-L1 adipocytes [3], although the inhibitors used in that study were somewhat less specific and potent than those used in the present study. Taken together, these data support our and others’ previous findings that in non-transformed cells and in a physiological context (i.e., in the presence of p110α protein), p110α is the principal PI3K isoform activated downstream of the insulin receptor [2,3].

### 2.2. Differential Interaction with Ras Does Not Confer PI3K Isoform Selectivity in Insulin Signalling

**Figure 3:**
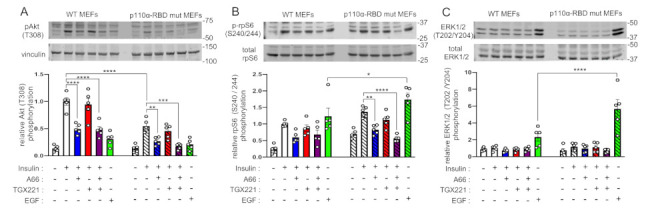
p110α maximal activation by insulin requires interaction with Ras. Wild-type (non-striped graph bars) and mutant (striped graph bars) p110α-RBD MEFs were treated with A66 (1 μM, blue graph bars) or TGX221 (0.5 μM, red graph bars) or a combination of both inhibitors (purple graph bars) followed by stimulation with 100 nM of insulin or with 15.6 nM of EGF (green graph bars) for 15 min at 37 °C. (**A**) Levels of Akt (T308) phosphorylation were determined by immunoblot analysis. Phosphorylation levels were normalised to vinculin used as a loading control. rpS6 (S240/244) phosphorylation (**B**) and ERK1/2 (T202/Y204) phosphorylation (**C**) were also detected in the same immunoblots. Representative immunoblots and corresponding bar graphs with pooled data from five (*n* = 5) independent experiments are shown. Data are presented as mean ± SEM. Statistical analysis was performed by two-way ANOVA with Dunnett’s multiple comparisons test between insulin-stimulated vehicle-treated and insulin-stimulated inhibitor-treated samples of the same genotype or Sidak’s multiple comparisons test between samples of different genotypes. * *p* < 0.05; ** *p* < 0.01; *** *p* < 0.001; **** *p* < 0.0001.

We next sought to examine whether interaction with Ras might account for PI3K isoform selectivity in insulin signalling. It has been shown before that the Ras-binding domain of p110β, unlike that of p110α, does not interact with Ras [20]. This offers a potential mechanism for the selectivity for p110α assuming that additional interaction of p110α with Ras might underlie the selective recruitment of p110α in IRS-1 complexes reported before [2]. In order to test this hypothesis, we used mouse embryonic fibroblasts (MEFs) isolated from mice with RBD mutations which abrogate binding of p110α to Ras [21]. We stimulated wild-type and mutant p110α-RBD MEFs with insulin and measured phosphorylation of Akt. As shown in Figure 3A, mutant p110α-RBD MEFs had a 50% decreased insulin-stimulated Akt phosphorylation compared to the wild-type, suggesting that a large part of insulin-stimulated activation of p110α occurs through interaction with Ras. However, the sensitivities of mutant p110α-RBD MEFs to inhibition of Akt phosphorylation by A66 and TGX221 were the same with those of wild-type MEFs. This suggests that interaction with Ras does not confer selectivity for p110α over p110β in recruitment to insulin receptor signalling complexes, otherwise impaired interaction of p110α with Ras would increase the contribution of p110β to insulin-stimulated Akt phosphorylation. Interestingly, unlike Akt phosphorylation, insulin-stimulated rpS6 phosphorylation tended to be higher in mutant p110α-RBD MEFs (Figure 3B). The same was the case between EGF-stimulated Akt and rpS6 phosphorylation. While EGF-stimulated Akt phosphorylation tended to be impaired in mutant p110α-RBD MEFs in line with a previous report [21], rpS6 phosphorylation was significantly higher. Notably, although insulin did not substantially stimulate ERK1/2 phosphorylation in these MEFs at the tested time point, EGF-stimulated ERK1/2 phosphorylation was significantly increased in mutant p110α-RBD MEFs (Figure 3C). This observation might be explained by PI3K p110α competition for Ras binding with other molecules (e.g., Raf) coupling Ras to MAP-kinase pathway activation.

### 2.3. p110α Is Preferentially Activated over p110β by β-AR in Adipocytes

**Figure 4:**
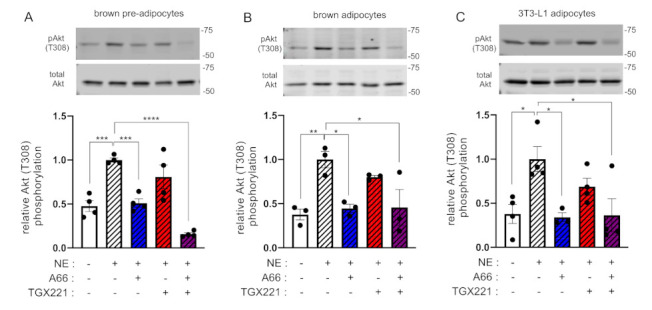
p110α is the principal PI3K isoform engaged in β-adrenergic signalling in adipocytes. Brown pre-adipocytes (**A**) or differentiated adipocytes (**B**) or differentiated 3T3-L1 adipocytes (**C**) were treated with A66 (1 μM, blue graph bars) or TGX221 (0.5 μM, red graph bars) or a combination of both inhibitors (purple graph bars) followed by stimulation with 1 μM of norepinephrine (NE, striped graph bars) for 15 min at 37 °C. Levels of Akt T308 phosphorylation were determined by immunoblot analysis. Phosphorylation levels were normalised to total Akt levels detected in a second blot performed in parallel using the same lysates. Representative immunoblots and corresponding bar graphs with pooled data from three or four (*n* = 3–4) independent experiments are shown. Data are presented as mean ± SEM. Statistical analysis was performed by one-way ANOVA with Dunnett’s multiple comparisons test between vehicle-treated NE-stimulated and all other samples. * *p* < 0.05; ** *p* < 0.01; *** *p* < 0.001; **** *p* < 0.0001.

We then sought to study the relative contributions of each of p110α and p110β to the adrenergic signalling pathway. We have reported before that the β-AR activates Akt through p110α [8]. However, we have not investigated the potential contribution of p110β to adrenergically stimulated Akt phosphorylation comparatively to p110α. We therefore used murine brown adipocytes and 3T3-L1 adipocytes and tested the effect of p110α- and p110β-selective inhibitors on Akt phosphorylation upon stimulation with the natural β2-AR agonist norepinephrine (NE). As shown in Figure 4, similar to insulin, NE-stimulated Akt phosphorylation was consistently sensitive only to A66, thus demonstrating that p110α is the principal PI3K isoform activated by β-AR in adipocytes.

### 2.4. Activation of PI3K Downstream of the β-AR Is Mainly Mediated by EPAC1

**Figure 5:**
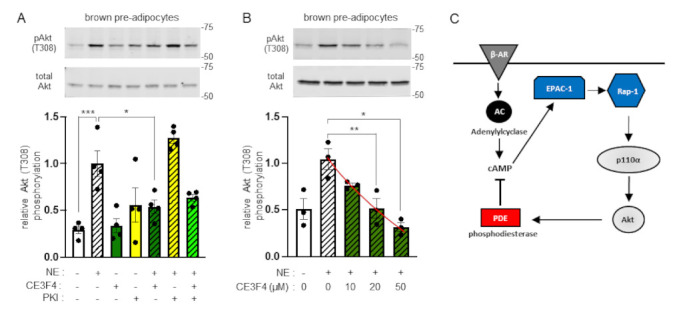
Activation of Akt downstream of β-AR is mediated by EPAC1. (**A**) Brown pre-adipocytes were treated with the EPAC1 inhibitor CE3F4 (20 μM, dark green graph bars) or the PKA inhibitor fragment (6-22) amide (PKI, 5 μM, yellow graph bars) or a combination of both inhibitors (bright green graph bar) followed by stimulation with 1 μM of norepinephrine (NE, striped graph bars) for 15 min at 37 °C. Levels of Akt T308 phosphorylation were determined by immunoblot analysis. Phosphorylation levels were normalised to total Akt levels detected in a second blot performed in parallel using the same lysates. A representative immunoblot and a bar graph with data from four (*n* = 4) independent experiments are shown. (**B**) Dose response of NE-stimulated Akt phosphorylation to CE3F4. Data are presented as mean ± SEM. Statistical analysis was performed by one-way ANOVA with Sidak’s multiple comparisons test between pre-determined pairs of treatments (**A**) or by repeated measures one-way ANOVA with Dunnett’s multiple comparisons test (**B**). * *p* < 0.05; ** *p* < 0.01; *** *p* < 0.001. (**C**) Schematic representation of p110α-dependent and Akt-mediated activation of PDE upon β-AR stimulation. According to the model, PI3K p110α and Akt are activated in the β-AR/cAMP pathway through EPAC1 and its target Rap1, as part of a feedback loop that downregulates cAMP levels through Akt-mediated PDE activation.

Unlike the well-studied mechanism of PI3K activation downstream of the insulin receptor, the pathway through which β-AR stimulates PI3K activity is unknown. β-AR stimulates adenylyl cyclase which results in elevation of cellular cAMP levels. We therefore surmised that one or both of the main cAMP effector molecules, protein kinase A (PKA) and guanine nucleotide exchange factor EPAC1 might mediate activation of PI3K downstream of the β-AR. We tested the effect of PKA and EPAC1 inhibitors, PKI-(6-22)-amide and CE3F4, respectively, on NE-stimulated Akt phosphorylation. As shown in Figure 5A,B, treatment of brown pre-adipocytes with the EPAC1 inhibitor CE3F4 reduced the NE-stimulated Akt phosphorylation in a dose-dependent manner indicating that PI3K activation downstream of the β-AR is mediated to a large extent by EPAC1. In contrast to EPAC1, inhibition of PKA increased the levels of NE-stimulated Akt activity, indicating that PKA might actually be supressing PI3K activation downstream of the β-AR (Figure 5A). EPAC1 is a GEF for Rap1, a member of the Ras family of small GTPases [22,23]. This provides a plausible mechanism of p110α activation through EPAC1, as Rap1 has been shown before to activate p110α [24]. We also tested the effect of siRNA-mediated EPAC1 knock-down on NE-stimulated Akt phosphorylation in brown pre-adipocytes. Treatment with siRNA reduced EPAC1 mRNA expression to 13% of the control levels (Figure S2). However, this treatment resulted to high basal levels of Akt phosphorylation causing a lower fold induction of Akt phosphorylation upon stimulation with NE (Figure S3). These data suggest that in the absence of the EPAC1 molecule, as opposed to inhibition of its enzymatic activity, and/or under prolonged downregulation of the EPAC1 molecule, other pathways might be able to compensate for its loss of function to some extent.

## 3. Discussion

In the present study we have revisited the question of the relative contribution of each of the ubiquitously expressed PI3K isoforms, p110α and p110β, in the insulin signalling pathway and we have also assessed the potential involvement of p110β in the β-AR signalling pathway. We have mainly used adipocytes in our experiments, as the adipose tissue is a key metabolic organ that regulates systemic insulin sensitivity and glucose homeostasis and adipocytes express high levels of both insulin receptors and β-ARs.

With regards to the relative contribution of each of the two isoforms to insulin receptor signalling, our experiments with the use of isoform-selective p110α and p110β inhibitors presented here are consistent with the previous reports that p110α is the principal isoform activated downstream of the insulin receptor [2,3]. Although other genetic studies have demonstrated involvement of p110β in insulin receptor signalling, this was either weak [4] or not dependent on its catalytic activity [5] or it was exerted in the absence of p110α in hepatocytes of mice with p110α gene deletion [6]. In fact, an earlier pharmacological study had already demonstrated that in addition to p110α, p110β and p110δ could be activated by insulin stimulation [19]. However, in non-transformed cells, such as 3T3-L1 fibroblasts and adipocytes, only p110α inhibition reduced insulin signalling, while a dependency to p110δ or p110β was only observed in hepatoma or in leukaemia cells.

As shown here, in brown adipocytes isolated from adipose tissue-specific p110α deleted mice, p110β can substitute for p110α in insulin-stimulated Akt phosphorylation in line with what was reported in hepatocytes lacking p110α [6]. An important point to note is that these data merely show that p110β can substitute for p110α in insulin-stimulated Akt phosphorylation. However, several reports have demonstrated that levels of Akt phosphorylation do not always correlate with Akt enzymatic activity [25,26] or with insulin sensitivity [27,28]. Therefore, unaffected insulin-stimulated Akt phosphorylation in p110α deficient cells, as a result of compensation by p110β, would not necessarily translate to normal insulin sensitivity. In this regard, we have recently reported that mice lacking p110α specifically in the adipose tissue, from which we have derived the brown adipocytes used in the present study, are severely insulin resistant despite the presence of p110β in adipocytes [8]. Furthermore, pharmacological studies of p110β-selective inhibitor administration in rodents do not support a physiological role of p110β in insulin signalling, as no effects on insulin sensitivity or glucose tolerance were seen at doses considered p110β-selective [29,30]. Collectively, these data support the notion that under physiological conditions, p110α is the principal PI3K isoform activated downstream of the insulin receptor.

Another outstanding issue that we have attempted to address with this study is the molecular mechanism of the selectivity for p110α in insulin signalling. We hypothesised that specific interaction with Ras might underlie the selective recruitment of p110α to IRS-1 complexes we have described before [2]. In this regard, Molinaro et al. have recently shown that virally mediated overexpression of dominant-negative Ras diminishes the level of p110α activation by insulin in hepatocytes [6]. Here, we have used embryonic fibroblasts from mice with mutated p110α-RBD and we have obtained similar results in that absolute levels of insulin-stimulated Akt phosphorylation were lower in mutant p110α-RBD MEFs. This finding was also consistent with earlier studies demonstrating that Ras is required for maximal activation of *Drosophila* PI3K [31]. However, our data do not support the hypothesis that p110α interaction with Ras underlies the selective recruitment of p110α in IRS-1 complexes, as the sensitivity of insulin-stimulated Akt phosphorylation to p110α or p110β inhibition in mutant p110α-RBD MEFs is similar to that in wild-type MEFs. This means that impaired interaction of p110α with Ras does not allow for compensation by p110β. This is, to some extent, similar to what has been reported for the selective recruitment of p110α over p110β in PDGF receptor signalling complexes [32]. Although a functional RBD was required for activation of p110β, but not of p110α, by PDGF, the RBD was not important for recruitment of either p110α or p110β to the PDGF-Receptor. Whether the observed difference in the level of insulin-stimulated Akt phosphorylation between wild-type and mutant p110α-RBD MEFs would translate to differences in insulin sensitivity and glucose tolerance would require metabolic phenotyping of mice with mutated p110α-RBD, which has not been reported so far.

In addition to the insulin pathway, we have also further dissected the molecular mechanism of PI3K activation downstream of the β-AR. In this regard, we have shown before that p110α is activated downstream of the β-AR [8]. In the present study we tested whether p110β contributes to Akt activation downstream of the β-AR. Similar to insulin stimulation, inhibition of p110α, but not of p110β, consistently reduced adrenergically stimulated Akt phosphorylation in both brown and white (3T3-L1) adipocytes. This demonstrates that p110α is the principal PI3K isoform activated downstream of the β-AR. By using an EPAC1 inhibitor, we found that this cAMP effector was required for stimulation of Akt phosphorylation by norepinephrine. These findings, together with our previously reported work [8], delineate a plausible mechanism (Figure 5C) for the activation of p110α downstream of the β-AR, as EPAC1 is a GEF for the Ras family member GTPase Rap1, which has previously been shown to stimulate the activity of p110α [24].

Overall, our data add further clarity in the roles of the two widely expressed PI3K isoforms p110α and p110β in insulin and adrenergic signalling pathways. This fundamental knowledge has important implications for therapeutic targeting of these isoforms in diseases, where effects on energy regulating pathways might affect the efficacy and safety of their inhibitors.

## 4. Materials and Methods

### 4.1. Reagents

PI3K p110α-selective inhibitor A66 (cat. no. 5595) was from Tocris/Bio-Techne (Minneapolis, MN, USA), PI3K p110β-selective inhibitor TGX221 (item no. 10007349), EPAC1 inhibitor CE3F4 (item no. 17767), and PKA inhibitor (PKI) fragment (2-22)-amide (item no. 17486) were from Cayman Chemical (Ann Arbor, MI, USA). All other reagents were purchased from Sigma-Aldrich (St. Louis, MO, USA) unless otherwise stated.

### 4.2. Cells, Culture and Differentiation

Isolation and immortalisation of wild-type and p110α^DEL^ brown pre-adipocytes has been described before [8]. Brown adipocyte differentiation was induced by treating confluent cell monolayers with 1 μg ml^−1^ insulin, 1 μM dexamethasone, 0.5 mM isobutylmethyxanthine (IBMX), 1 μM rosiglitazone (Cayman Chemical) and 1 nM 3,3′,5-Triiodo-l-thyronine (T3). After 48 h, the medium was replaced with fresh DMEM (Gibco, Thermo Fisher Scientific, Waltham, MA, USA) supplemented with 10% foetal bovine serum (Gibco, ThermoFisher Scientific). Cell monolayers were used in experiments upon completion of differentiation, typically 1 week after induction.

3T3-L1 mouse fibroblasts were obtained from ATCC and maintained in DMEM with 4.5 g/L glucose supplemented with 10% newborn calf serum (Gibco, ThermoFisher Scientific) at 37 °C in a humidified incubator with 5% CO_2_. For 3T3-L1 adipocyte differentiation, cells were allowed to reach confluence. 48  h post-confluence, cell culture medium was replaced with differentiation induction medium consisting of DMEM supplemented with 10% foetal bovine serum, 0.5 mM isobutylmethylxanthine, 1 μM dexamethasone, 1 μM rosiglitazone and 1 μg/mL bovine insulin. 48 h after induction of differentiation, the medium was replaced with DMEM supplemented with 10% FBS and 1 μg/mL insulin. After a further 48 h, the medium was replaced with DMEM supplemented with 10% FBS. Complete differentiation was typically reached by day 8.

Hepa 1-6 murine hepatoma cells were obtained from ATCC. Mouse embryonic fibroblast (MEFs) from mice with mutated p110α-RBD (T208D and K227A) [21] and wild-type counterparts were provided by Prof. Julian Downward (The Crick Institute, London, UK). MEFs derived from mice with a p110β kinase-dead (D931A) mutation [33] were provided by Prof. Bart Vanhaesebroeck (UCL Cancer Institute, London, UK).

### 4.3. Inhibitor Treatments, Cell Stimulation, Lysis and Immunoblot Analysis

Cells were serum deprived in DMEM supplemented with 0.2% fatty acid-free bovine serum albumin for 3 h prior to cell stimulation. Inhibitors or vehicle (DMSO) were added at the concentrations indicated in the respective figure legends for the last 30 min of the serum deprivation period. At the end of the serum deprivation period, cells were stimulated at the doses and for the times indicated at 37 °C followed by shifting on ice, aspiration of media and rinsing with ice-cold phosphate buffered saline. Cell monolayers were then homogenised in a lysis buffer containing 50 mM Tris-HCl pH 7.4, 100 mM NaCl, 50 mM NaF, 5 mM EDTA, 2 mM EGTA, 40 mM beta-glycerophosphate, 10 mM sodium pyrophosphate, 1% Triton X-100 and protease inhibitors. Proteins were separated by SDS-PAGE and then transferred to PVDF membrane (Immobilon-FL, Millipore, Burlington, MA, United States). 5% non-fat milk was used to saturate the membrane prior to incubation with primary antibodies (all diluted at 1:1000). p110α (cat. no. 4249), p110β (cat. no. 3011), pT308 Akt (cat. no. 2965), total Akt (cat. no. 9272), pS240/244 rpS6 (cat. no. 5364), total rpS6 (cat. no. 2317), pT202/Y204 ERK1/2 (cat. no. 4370), total ERK1/2 (cat. no. 4696) antibodies were from Cell Signaling Technology (CST, Danvers, MA, USA). Vinculin antibody (cat. no. V9264, diluted 1:10,000) was from Sigma. Detection was performed with fluorescently labelled secondary antibodies (anti-mouse DyLight 800-conjugated, Rockland, Limerick, PA, USA; cat. no. 610-145-002, diluted 1:5000 and anti-rabbit Alexa-Fluor 680-conjugated, Invitrogen/Thermo Fisher Scientific, cat. no. A21076, diluted 1:5000), using an Odyssey CLx infrared scanner (LI-COR Biosciences, Lincoln, NE, USA). Detection of PI3K p110α and p110β was performed with HRP-conjugated, goat anti-Rabbit IgG (cat. no. P0448, DAKO-Agilent, Santa Clara, CA, USA) diluted 1:2000 and radiographic film detection using ECL reagent (GE Healthcare, Buckinghamshire, UK). Quantification was performed using Image Studio Lite Ver 5.2 (LI-COR Biosciences). Original, uncropped images of the blots are shown in Supporting Information.

### 4.4. siRNA-Mediated EPAC1 Knockdown

EPAC1 knockdown in brown pre-adipocytes was performed by reverse transfection with EPAC1 siRNA (sense: 5′-CCACAGAGCAUGUGCACAA(TT)-3′ and antisense: 5′-UUGUGCACAUGCUCUGUGG(TG)-3′, purchased from Eurofins) using Lipofectamine RNAiMax (Invitrogen/Thermo Fisher Scientific). Briefly, 3 μL of Lipofectamine RNAiMax were mixed with siRNA (10 nM final concentration) in 0.5 mL OPTI-MEM (Gibco/Thermo Fisher Scientific) and added in 6-well dishes. After 20 min, 36,000 cells, suspended in cell culture medium free of antibiotics, were added to each well and the dishes were placed in the cell culture incubator. Cells were used in experiments 72 h after siRNA transfection.

The efficiency of EPAC1 knockdown was determined by measuring EPAC1 mRNA levels by RT-PCR. Briefly, total RNA was extracted from pre-adipocytes using TRI reagent (Sigma) 72 h after siRNA transfection. RNA was reversed transcribed to cDNA using Omniscript RT kit (Qiagen, Hilden, Germany) and an oligo-dT primer according to the manufacturer’s instructions. cDNA was used as a template for PCR for EPAC1 (using primers 5′-TCTTACCAGCTAGTGTTCGAGC-3′ and 5′-AATGCCGATATAGTCGCAGATG-3′). Actin-α1 was also amplified as a loading control (using primers 5′-GGTGTCATGGTAGGTATGGGT-3′ and 5′-CGCACAATCTCACGTTCAG-3′). PCR products were resolved on an 1.8% agarose gel containing ethidium bromide. Signal intensity of the amplicons was quantified in digital images of the gels using Image Studio Lite Ver 5.2 (LI-COR Biosciences). Signal intensity of the EPAC1 amplicon was normalised to the intensity of the corresponding actin amplicon.

### 4.5. Glucose Uptake Assay

3T3-L1 adipocytes grown and differentiated in 6-well plates as described above were cultured in serum-free DMEM supplemented with 0.2% bovine serum albumin for 3 h followed by incubation in glucose-free Krebs Ringer Bicarbonate Buffer (30 mM Hepes-NaOH pH 7.4, 10 mM NaHCO_3_, 120 mM NaCl, 4 mM KH_2_PO_4_, 1 mM MgSO_4_, 0.75 mM CaCl_2_) in the presence or absence of PI3K inhibitors for 30 min. Cells were stimulated with 100 nM insulin for 20 min followed by addition of 10 mM ‘cold’ 2-deoxy-D-glucose and 5 μCi of [^3^H]-2-deoxy-D-glucose (Perkin Elmer, Waltham, MA, USA). After 10 min, plates were shifted on ice and rinsed three times with ice-cold phosphate buffered saline. Cells were lysed in 0.5 mL 0.5 N NaOH/0.1% SDS for 30min at 37 °C followed by neutralisation with 0.5 mL 0.5 N HCl. Radioactive incorporation was then measured by scintillation counting. Non-specific radioactive incorporation was measured in the presence of 20 μM cytochalasin-B and subtracted from the test samples’ radioactivity counts.

### 4.6. Statistical Analysis

Data are presented as mean ± standard error of the mean (SEM). Statistical analyses were performed with Prism 8 (GraphPad, San Diego, CA, USA). Number of biological repeats and specific statistical tests for each experiment are detailed in the respective figure legends. Statistical significance is indicated with asterisks: * *p* < 0.05; ** *p* < 0.01; *** *p* < 0.001; **** *p* < 0.0001.

## Data Availability

The data that support the findings of this study are included in this published article and its Supplementary Materials files. All other relevant data are available on request from the corresponding author.

## Supplementary Materials

The following are available online at https://www.mdpi.com/article/10.3390/ijms222312813/s1.
