# Supplementary material for: Dominant Role of PI3K p110α over p110β in Insulin and β-Adrenergic Receptor Signalling
Source: Int J Mol Sci. 2021 Nov 26;22(23):12813. doi: 10.3390/ijms222312813 (PMC8657683; doi:10.3390/ijms222312813)

Supporting Information

Zhang et al. ‘Dominant role of PI3K p110α over p110β in insulin and β-adrenergic receptor signalling'

This file contains Supplementary Figures S1-S3 and original, uncropped blots for Figures 1-5 and S1-S3.

Figure S1

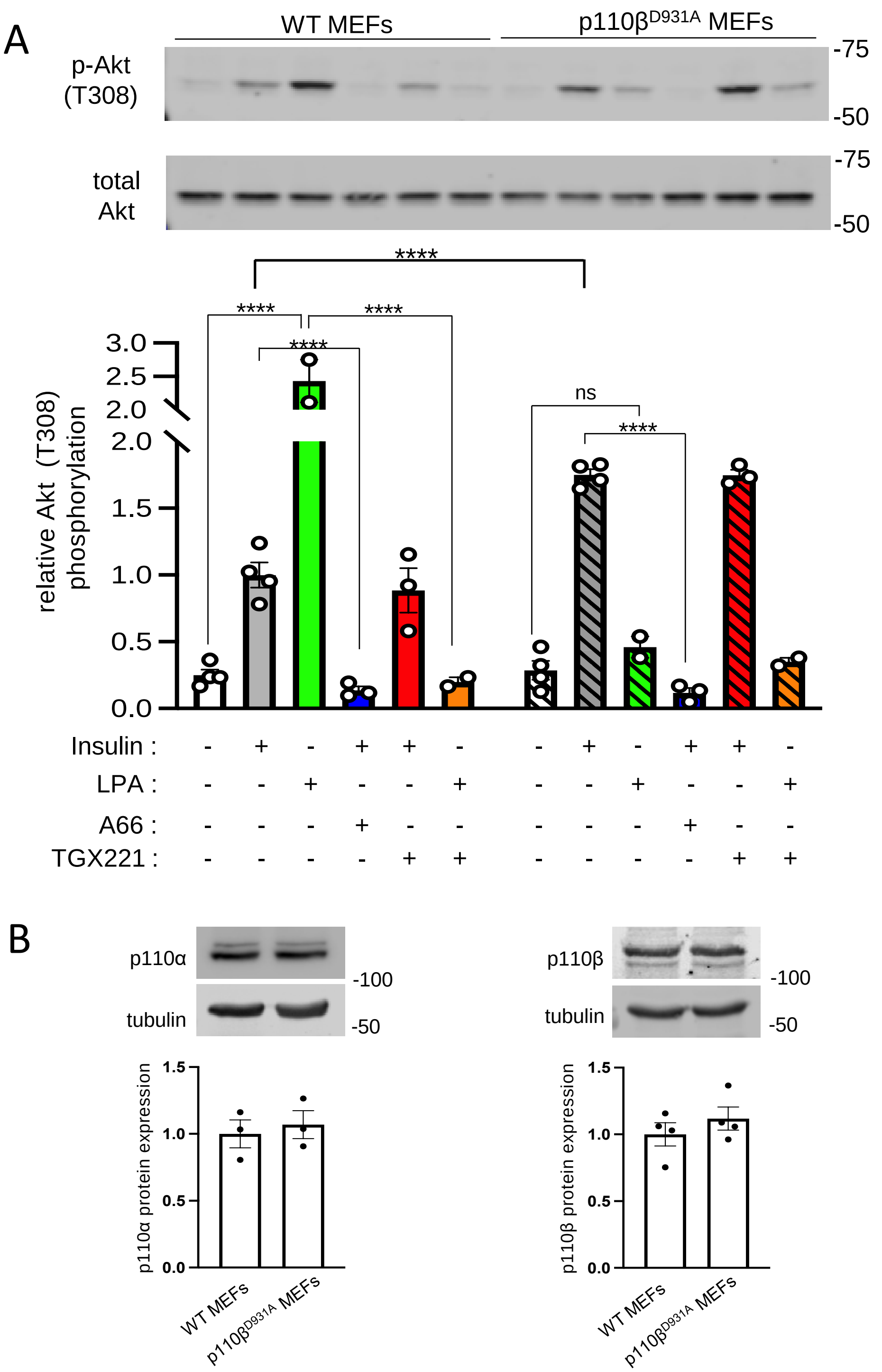

Figure S1 p110α is the principal PI3K isoform engaged in insulin signalling in MEFs. Wild-type (WT) and p110β^{D931A} MEFs were treated with A66 (1 μM) or TGX221 (0.5 μM) or a combination of both inhibitors followed by stimulation with 10 nM of insulin for 15 min at 37°C. (**A**) Levels of Akt (T308) phosphorylation were determined by immunoblot analysis. Phosphorylation levels were normalised to total Akt levels detected in a second blot performed in parallel using the same lysates. Treatment with LPA (10 μM), which is known to activate Akt via p110β, was used as a control of lack of p110β activity in p110β^{D931A} MEFs (**B**) The levels of expression of p110α and p110β in WT and p110β^{D931A} MEFs were also determined by immunoblot analysis. p110α and p110β signal intensities were normalised to tubulin. Representative immunoblots and corresponding bar graphs with data from three or four (n=3-4) independent experiments are shown. Data are presented as mean ± SEM. Statistical analysis was performed by two-way ANOVA with Sidak's multiple comparisons test (**A**) or by unpaired two-tailed t-test (**B**).

Figure S2

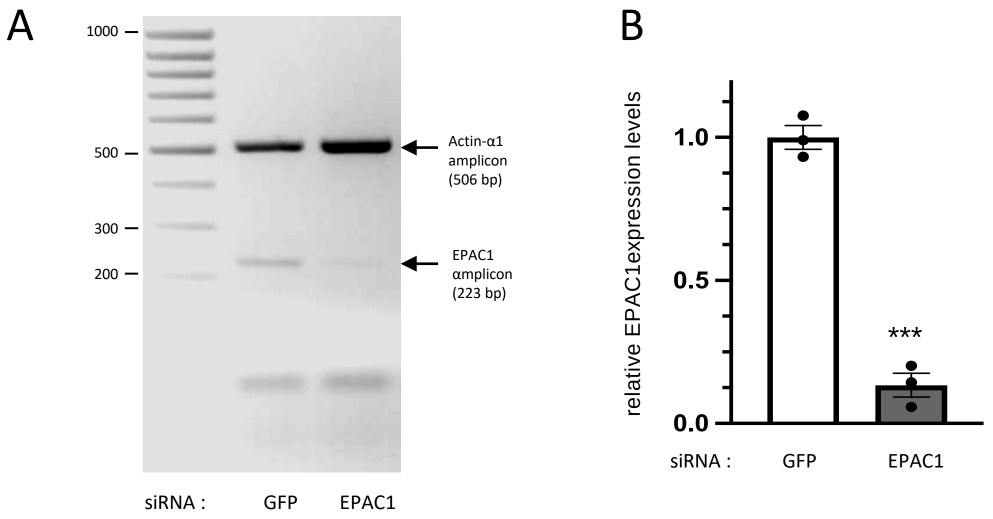

Figure S2 siRNA-mediated EPAC1 knock-down in brown pre-adipocytes. Levels of EPAC1 mRNA were determined 72 h following transection of siRNAs targeting either GFP (control) or EPAC1 by RT-PCR and densitometric quantification of the EPAC1 amplicon's intensity following agarose gel electrophoresis. Actin-α1 amplification was used as a loading control (A). (B) Average EPAC1 mRNA expression upon knock-down with EPAC1 siRNA was 13% of the control (GFP siRNA). Data are derived from three independent experiments and presented as mean ± SEM. Statistical analysis was performed by unpaired two-tailed t-test.

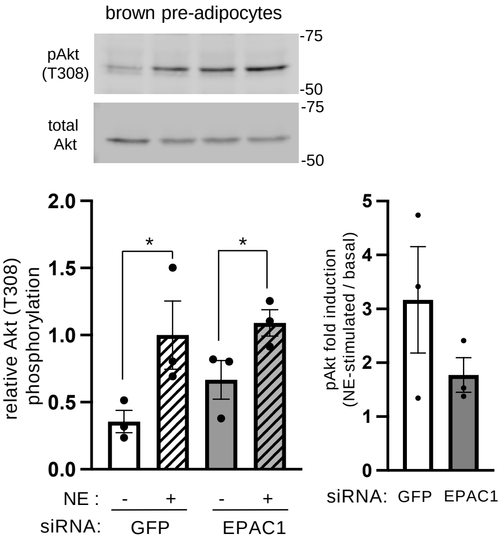

Figure S3 EPAC1 siRNA-mediated knockdown reduces NE-stimulated Akt fold activation. Brown pre-adipocytes were transfected with the EPAC1 siRNA or GFP esiRNA control. 72 h later, cells were serum-deprived for 3h followed by stimulation with 1 μM norepinephrine (NE) for 15 min at 37ºC. Levels of Akt T308 phosphorylation were determined by immunoblot analysis. Phosphorylation levels were normalised to total Akt levels detected in a second blot performed in parallel using the same lysates. A representative immunoblot and a bar graph with data from three (n=3) independent experiments are shown. Values from the bar graph have been used for calculation of NE-stimulated Akt phosphorylation fold induction shown in the right-hand bar graph. Data are presented as mean ± SEM. Statistical analysis was performed by two-way ANOVA with Tukey's multiple comparisons test.

Figure 1A: Brown pre-adipocytes pAkt (T308) blot

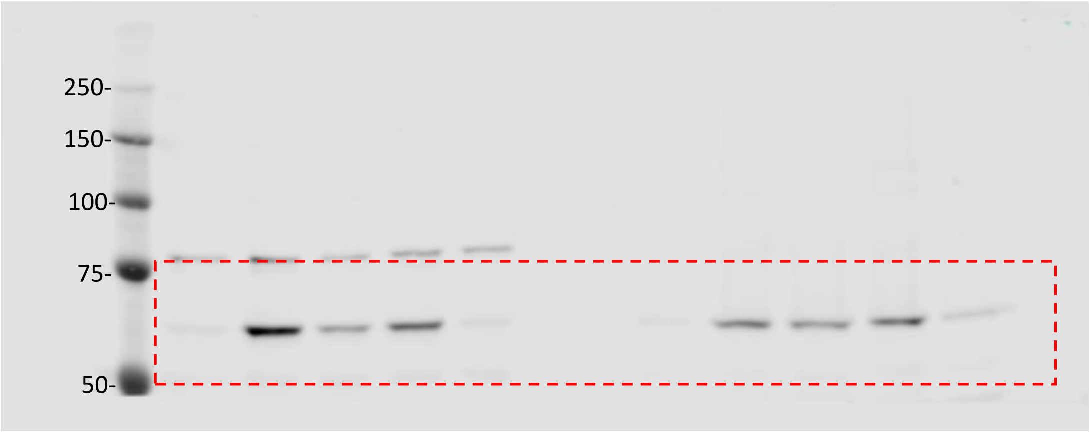

Figure 1A: Brown pre-adipocytes total Akt blot

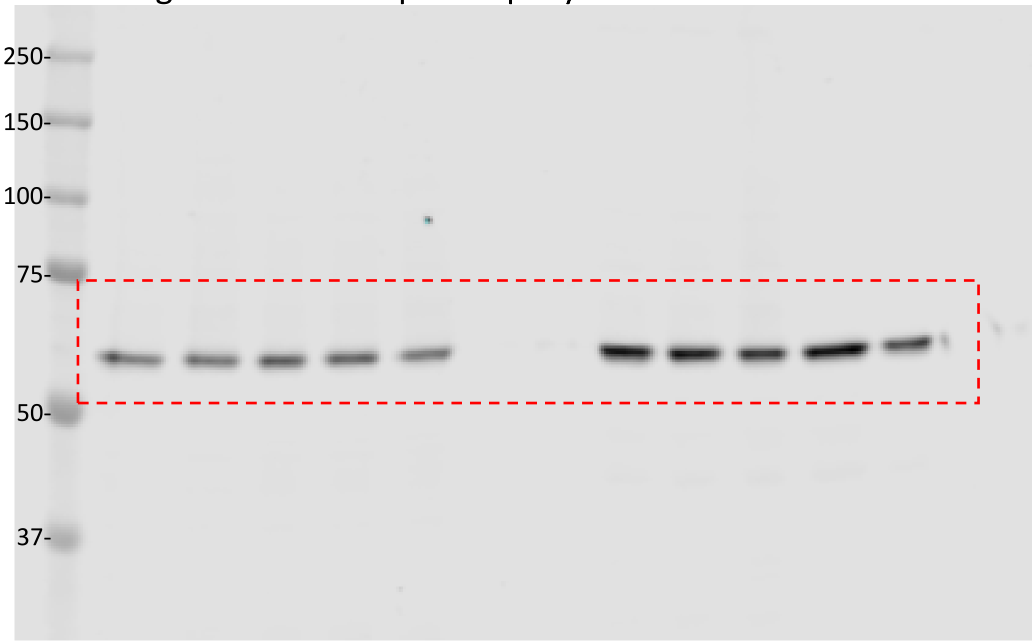

Figure 1B: Brown pre-adipocytes p-rpS6 (S240/244) blot

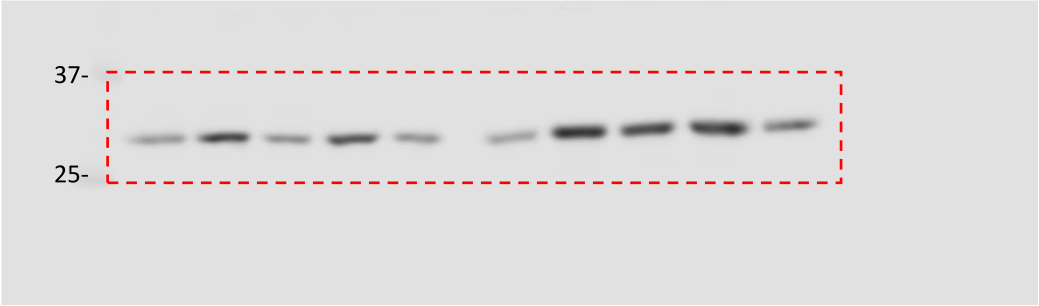

Figure 1B: Brown pre-adipocytes total rpS6 blot

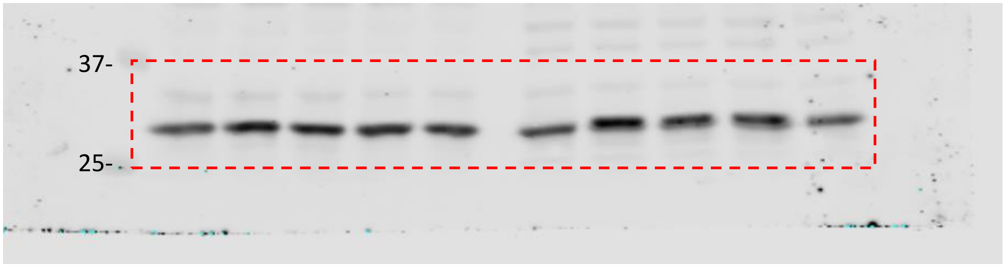

Figure 1C: Brown pre-adipocytes p110α blot

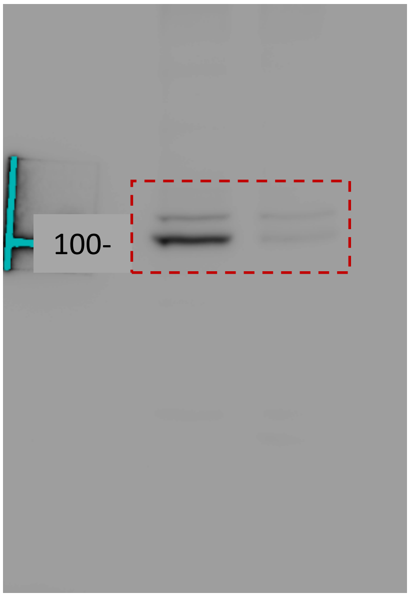

Brown pre-adipocytes vinculin blot for p110α

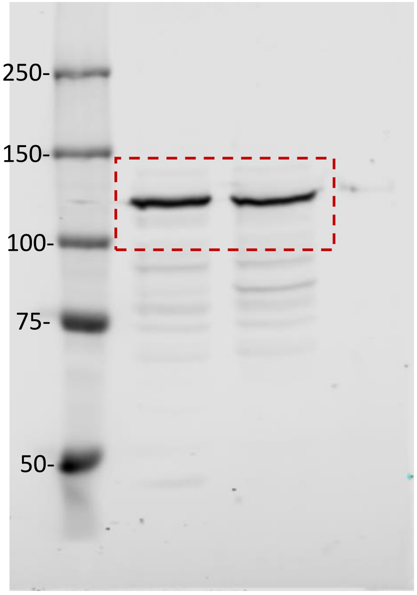

Figure 1C: Brown pre-adipocytes p110β blot

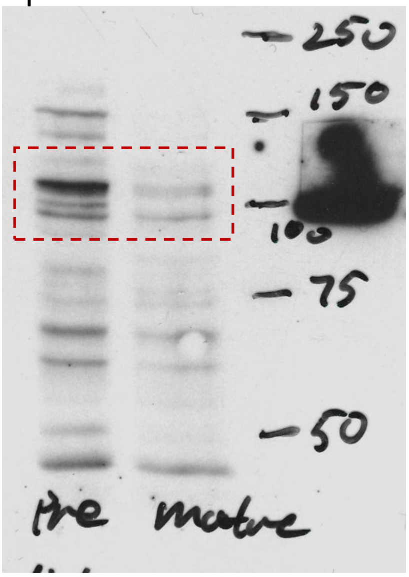

Brown pre-adipocytes vinculin blot for p110β

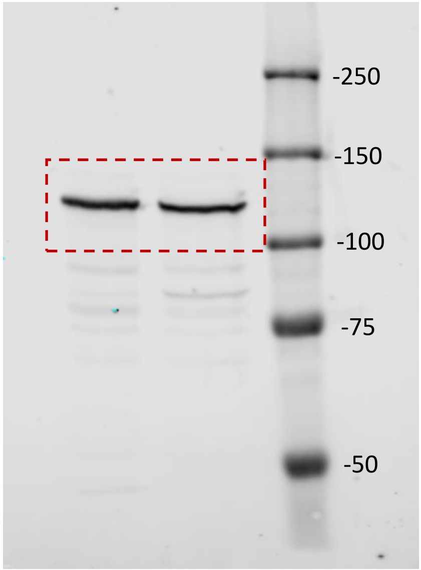

Figure 1D: Brown adipocytes pAkt blot

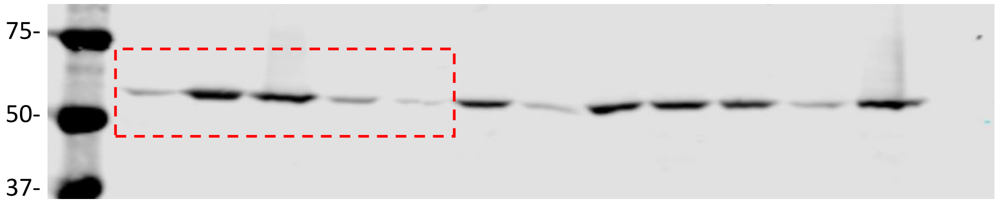

Figure 1D: Brown adipocytes total Akt blot

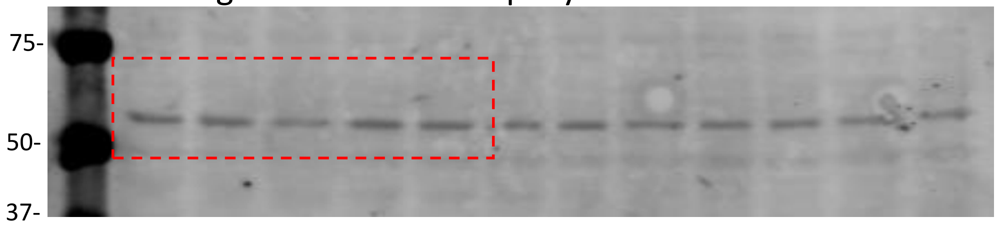

Figure 2A: 3T3-L1 pre-adipocytes pAkt (T308) blot

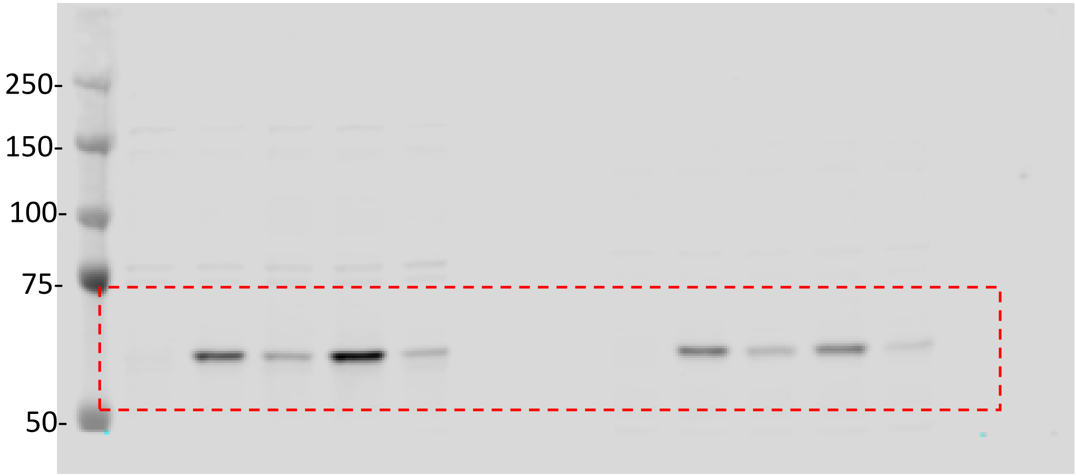

Figure 2A: 3T3-L1 pre-adipocytes total Akt blot

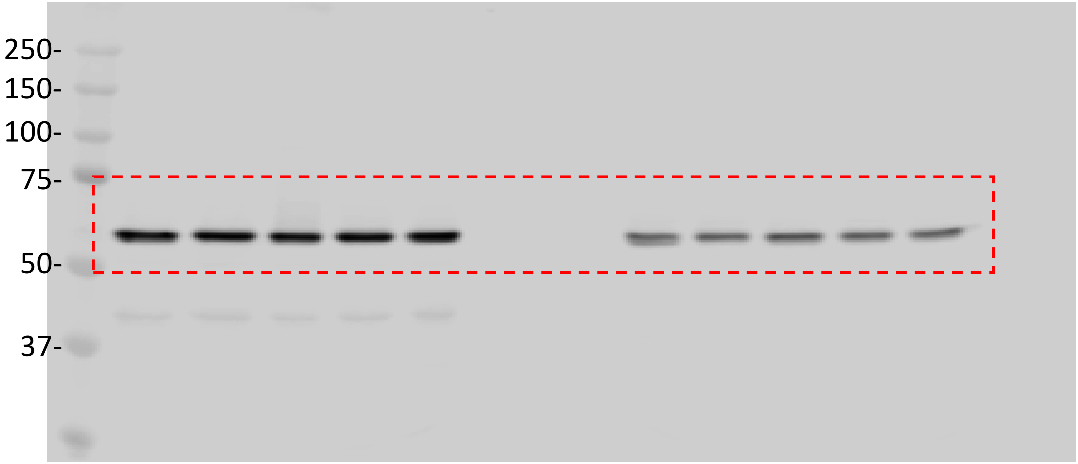

Figure 2B: 3T3-L1 pre-adipocytes p-rpS6 (S240/244) blot

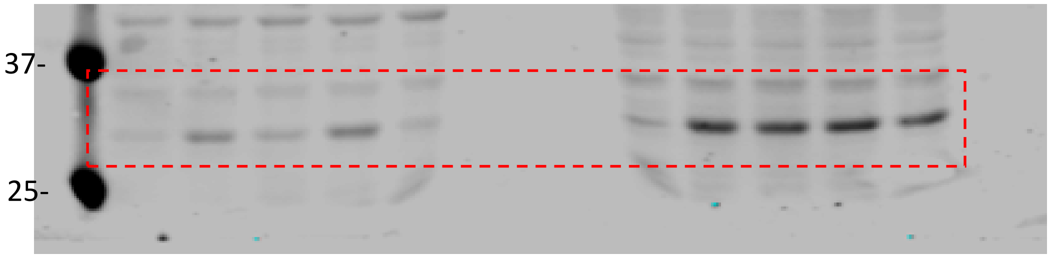

Figure 2B: 3T3-L1 pre-adipocytes total rpS6 blot

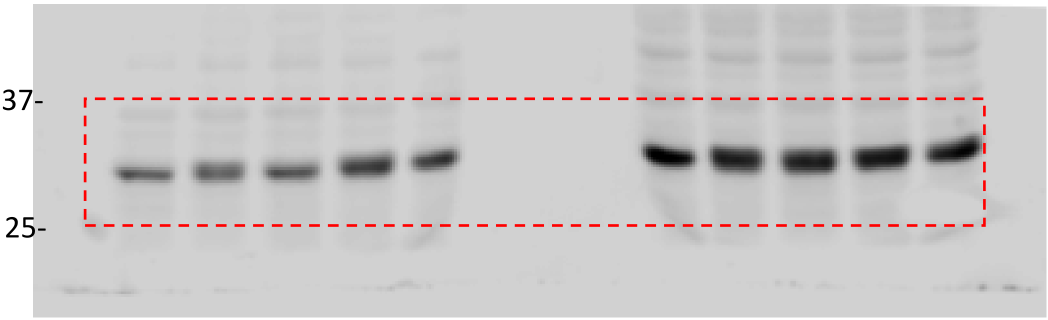

Figure 2C:3T3-L1 pre-adipocytes p110α blot

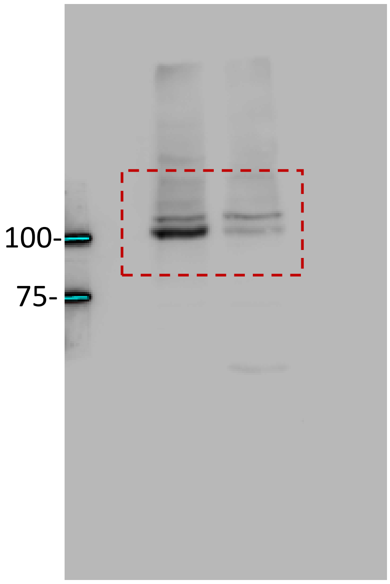

3T3-L1 pre-adipocytes vinculin blot for p110α

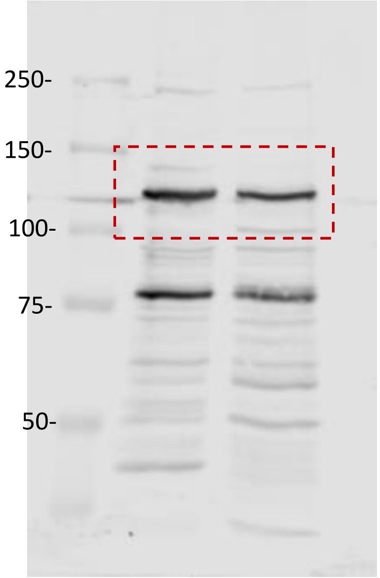

Figure 2C: 3T3-L1 pre-adipocytes p110β blot

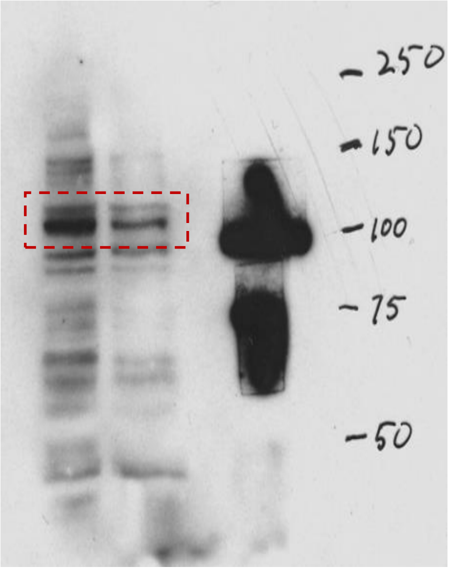

3T3-L1 pre-adipocytes vinculin blot for p110β

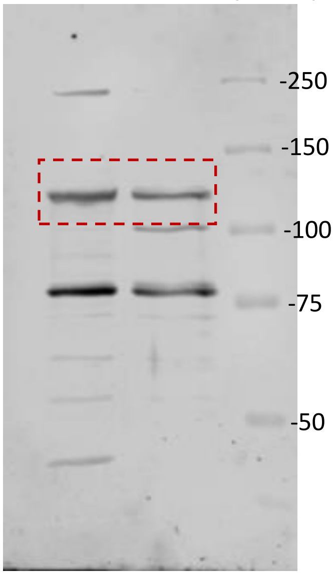

Figure2D: Hepa 1-6 pAkt blot

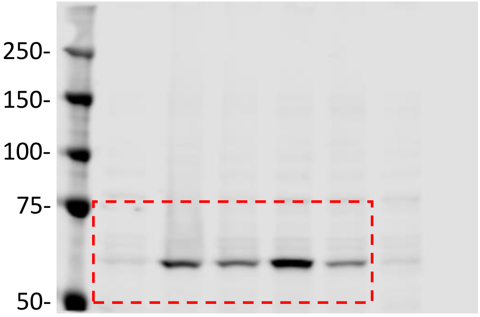

Figure2D: Hepa 1-6 total Akt blot

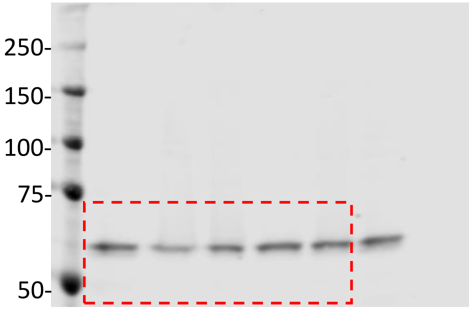

Figure2D: Hepa 1-6 p-rpS6 blot

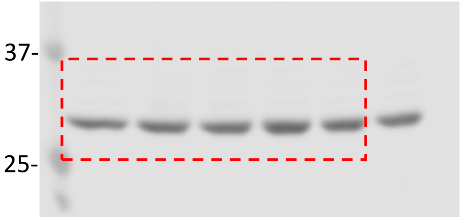

Figure2D: Hepa 1-6 total rpS6 blot

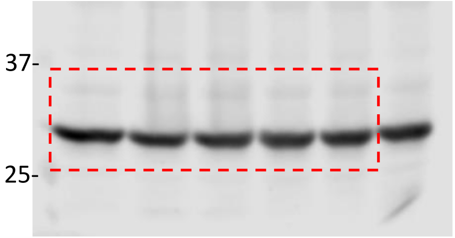

Figure 3A: p110α-RBD-MEFs pAkt blot

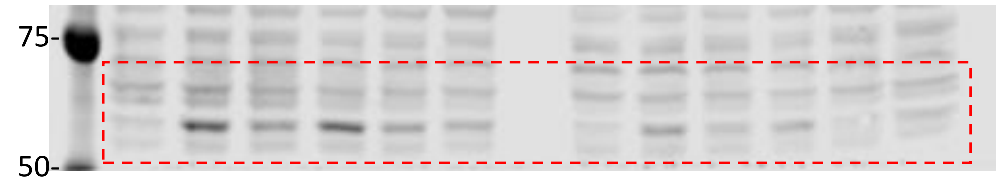

Figure 3A: p110α-RBD-MEFs vinculin blot

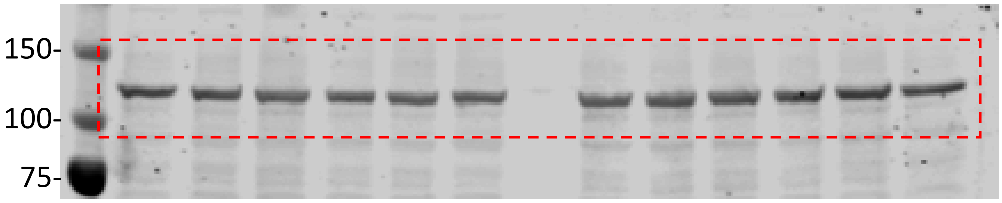

Figure 3B: p110α-RBD-MEFs p-rpS6 blot

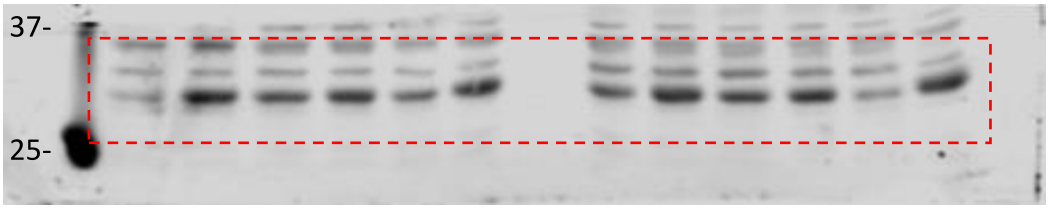

Figure 3B: p110α-RBD-MEFs total rpS6 blot

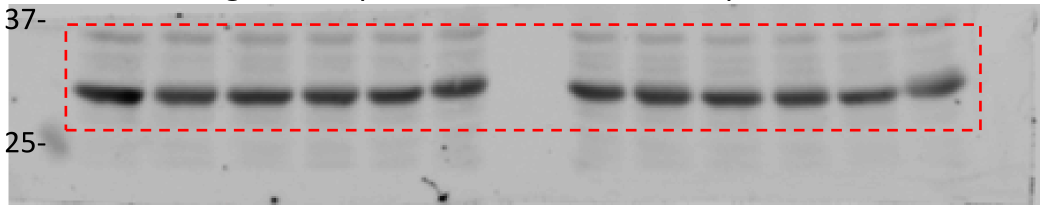

Figure 3C: p110α-RBD-MEFs pERK1/2 blot

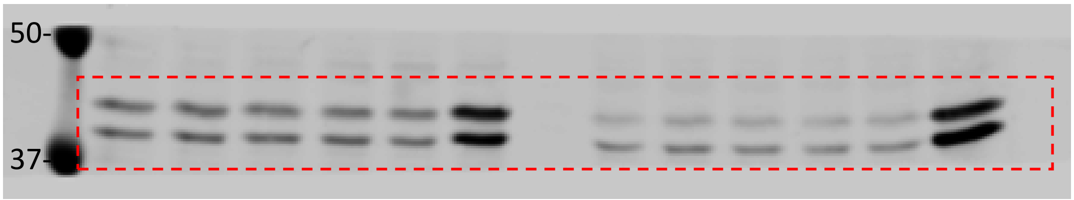

Figure 3C: p110α-RBD-MEFs total ERK1/2 blot

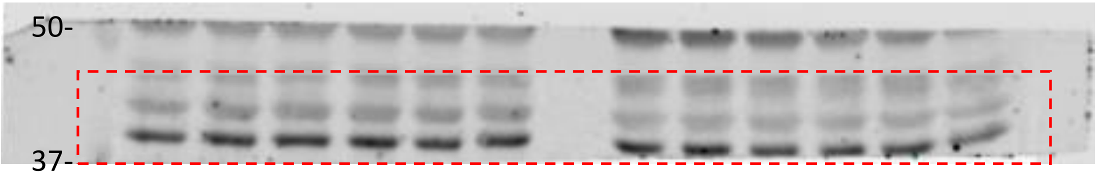

Figure 4A: Brown pre-adipocytes pAkt blot

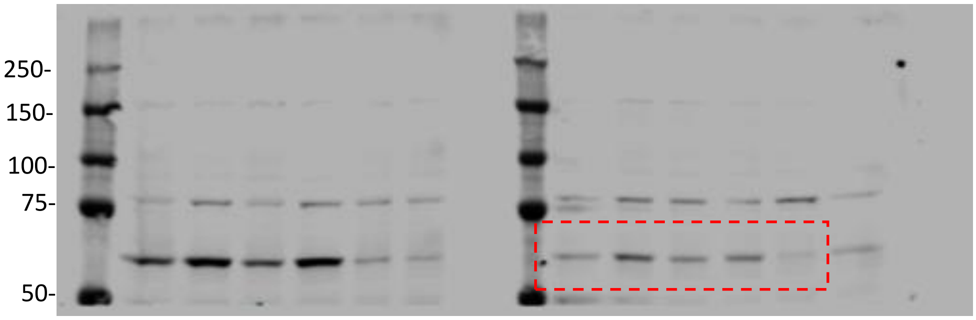

Figure 4A: Brown pre-adipocytes total Akt blot

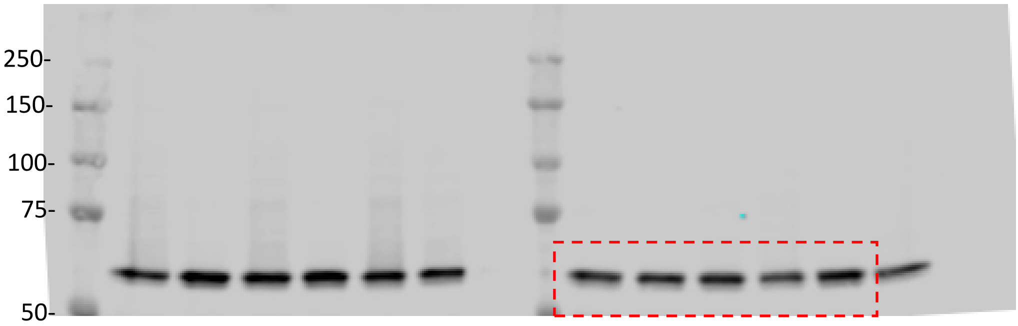

Figure 4B: Brown adipocytes pAkt blot

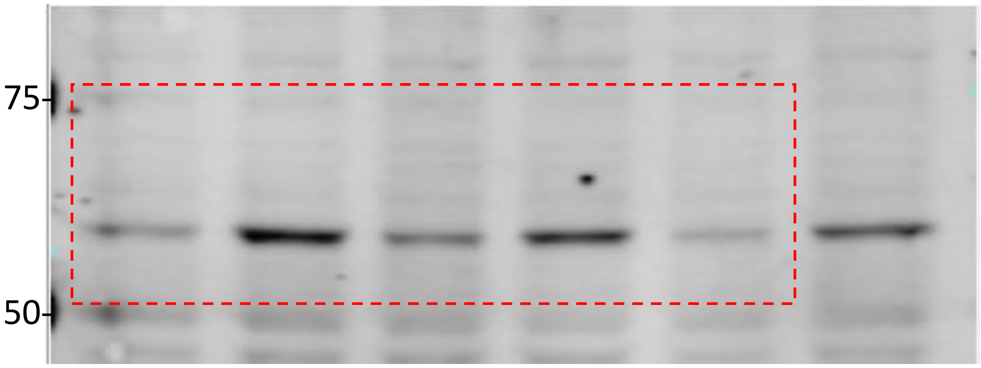

Figure 4B: Brown adipocytes total Akt blot

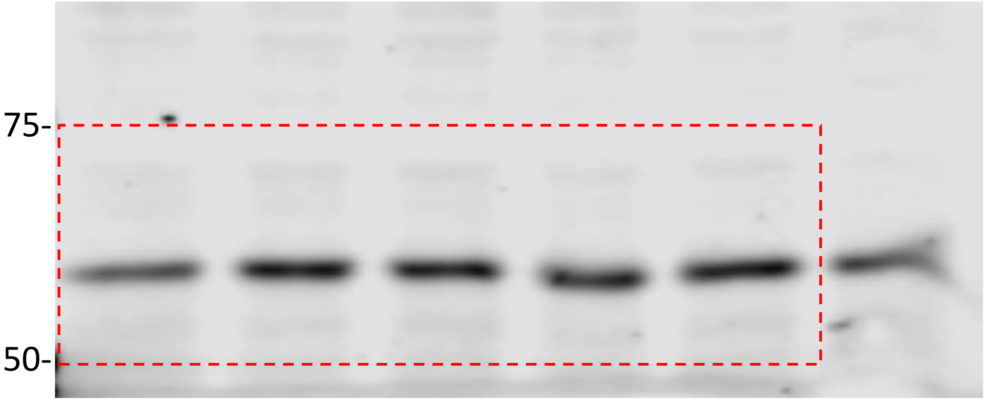

Figure 4C: 3T3-L1 adipocytes pAkt blot

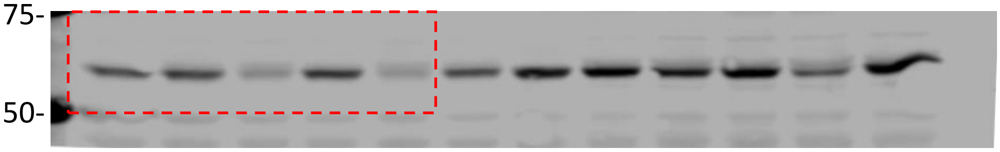

Figure 4C: 3T3-L1 adipocytes total Akt blot

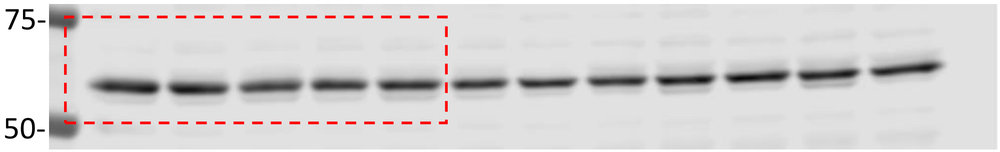

Figure 5A: Brown pre-adipocytes pAkt (T308) blot

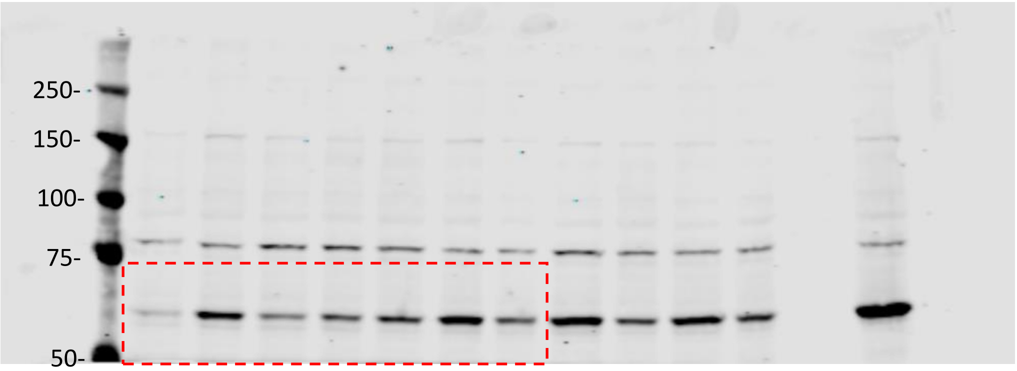

Figure 5A: Brown pre-adipocytes total Akt blot

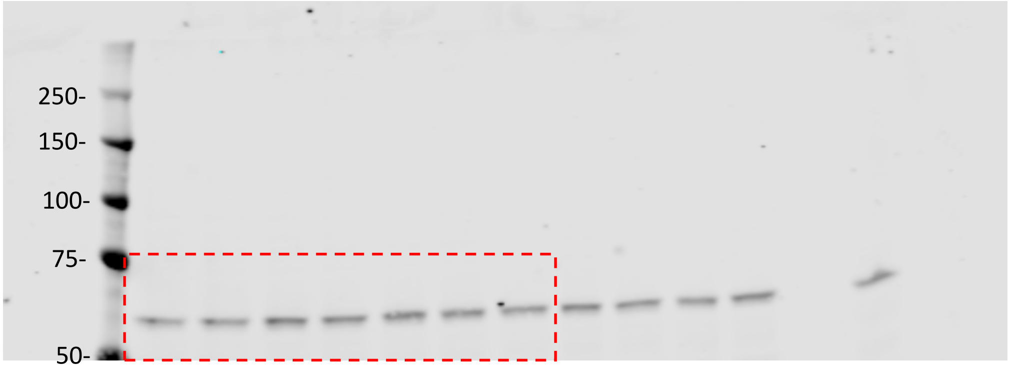

Figure 5B: Brown pre-adipocytes pAkt (T308) blot

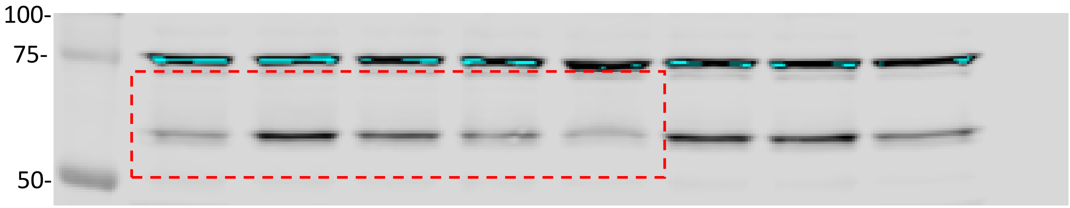

Figure 5B: Brown pre-adipocytes total Akt blot

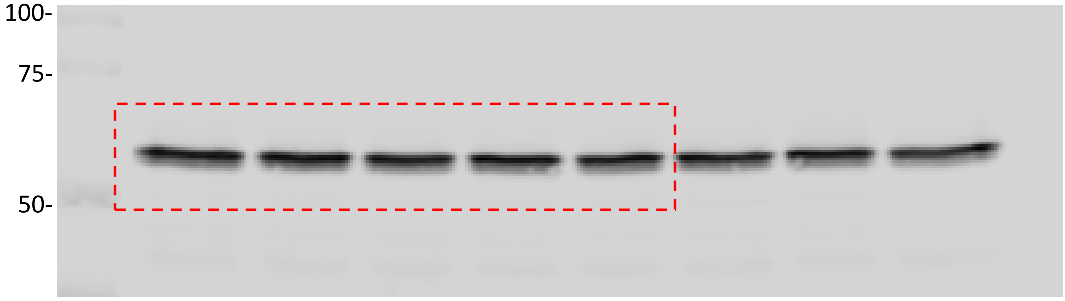

Figure S1: p110β^{D931A} MEFs pAkt (T308) blot

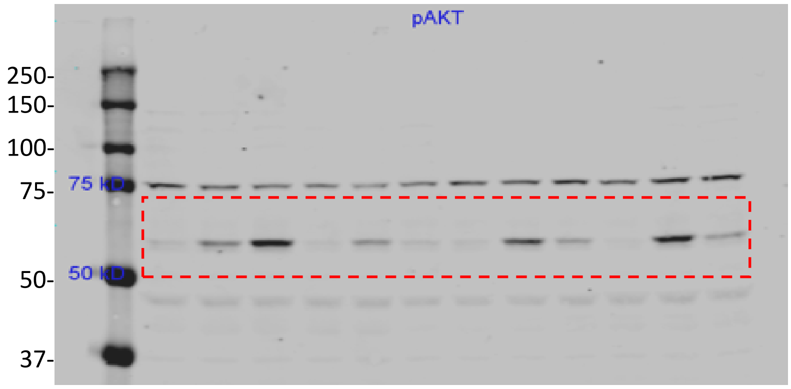

Figure S1: p110β^{D931A} MEFs total Akt blot

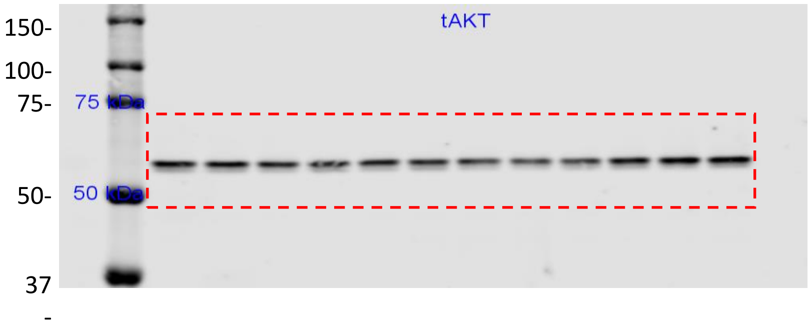

Figure S1: p110β^{D931A} MEFs p110α blot

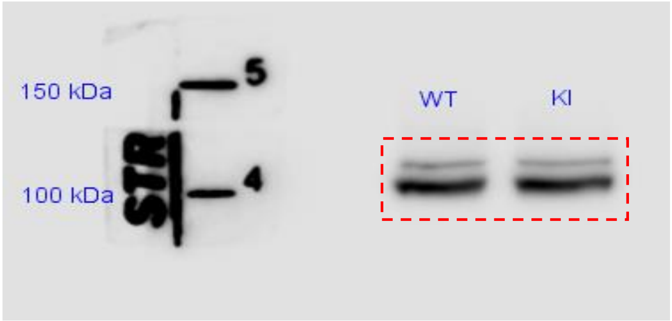

Figure S1: tubulin probing for p110α blot

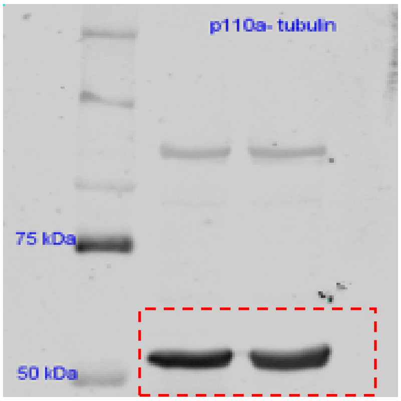

Figure S1: p110β^{D931A} MEFs p110β blot

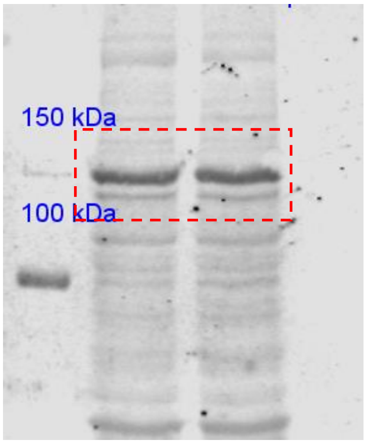

Figure S1: tubulin probing for p110β blot

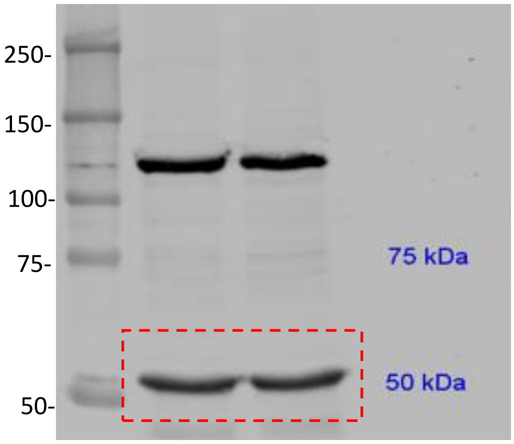

Figure S3: Brown pre-adipocytes pAkt (T308) blot

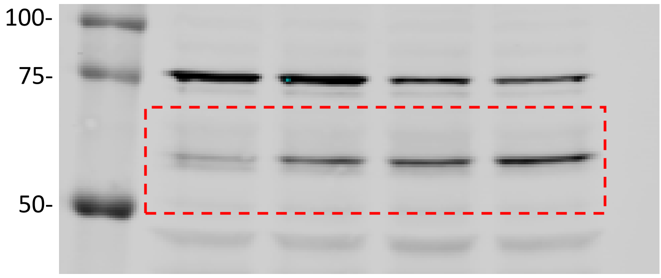

Figure S3: Brown pre-adipocytes total Akt blot

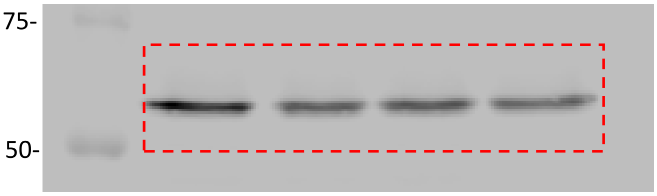

Supplement: Supplementary file 1 [file ijms-22-12813-s001.zip › ijms-949664-supplementary.pdf]
